# KRAGEN: a knowledge graph-enhanced RAG framework for biomedical problem solving using large language models

**DOI:** 10.1093/bioinformatics/btae353

**Published:** 2024-06-03

**Authors:** Nicholas Matsumoto, Jay Moran, Hyunjun Choi, Miguel E Hernandez, Mythreye Venkatesan, Paul Wang, Jason H Moore

**Affiliations:** Department of Computational Biomedicine, Center for Artificial Intelligence Research and Education, Cedars Sinai Medical Center, West Hollywood, CA 90069, United States; Department of Computational Biomedicine, Center for Artificial Intelligence Research and Education, Cedars Sinai Medical Center, West Hollywood, CA 90069, United States; Department of Computational Biomedicine, Center for Artificial Intelligence Research and Education, Cedars Sinai Medical Center, West Hollywood, CA 90069, United States; Department of Computational Biomedicine, Center for Artificial Intelligence Research and Education, Cedars Sinai Medical Center, West Hollywood, CA 90069, United States; Department of Computational Biomedicine, Center for Artificial Intelligence Research and Education, Cedars Sinai Medical Center, West Hollywood, CA 90069, United States; Department of Computational Biomedicine, Center for Artificial Intelligence Research and Education, Cedars Sinai Medical Center, West Hollywood, CA 90069, United States; Department of Computational Biomedicine, Center for Artificial Intelligence Research and Education, Cedars Sinai Medical Center, West Hollywood, CA 90069, United States

## Abstract

**Motivation:**

Answering and solving complex problems using a large language model (LLM) given a certain domain such as biomedicine is a challenging task that requires both factual consistency and logic, and LLMs often suffer from some major limitations, such as hallucinating false or irrelevant information, or being influenced by noisy data. These issues can compromise the trustworthiness, accuracy, and compliance of LLM-generated text and insights.

**Results:**

Knowledge Retrieval Augmented Generation ENgine (KRAGEN) is a new tool that combines knowledge graphs, Retrieval Augmented Generation (RAG), and advanced prompting techniques to solve complex problems with natural language. KRAGEN converts knowledge graphs into a vector database and uses RAG to retrieve relevant facts from it. KRAGEN uses advanced prompting techniques: namely graph-of-thoughts (GoT), to dynamically break down a complex problem into smaller subproblems, and proceeds to solve each subproblem by using the relevant knowledge through the RAG framework, which limits the hallucinations, and finally, consolidates the subproblems and provides a solution. KRAGEN’s graph visualization allows the user to interact with and evaluate the quality of the solution’s GoT structure and logic.

**Availability and implementation:**

KRAGEN is deployed by running its custom Docker containers. KRAGEN is available as open-source from GitHub at: https://github.com/EpistasisLab/KRAGEN.

## 1 Introduction

**Figure 1.**
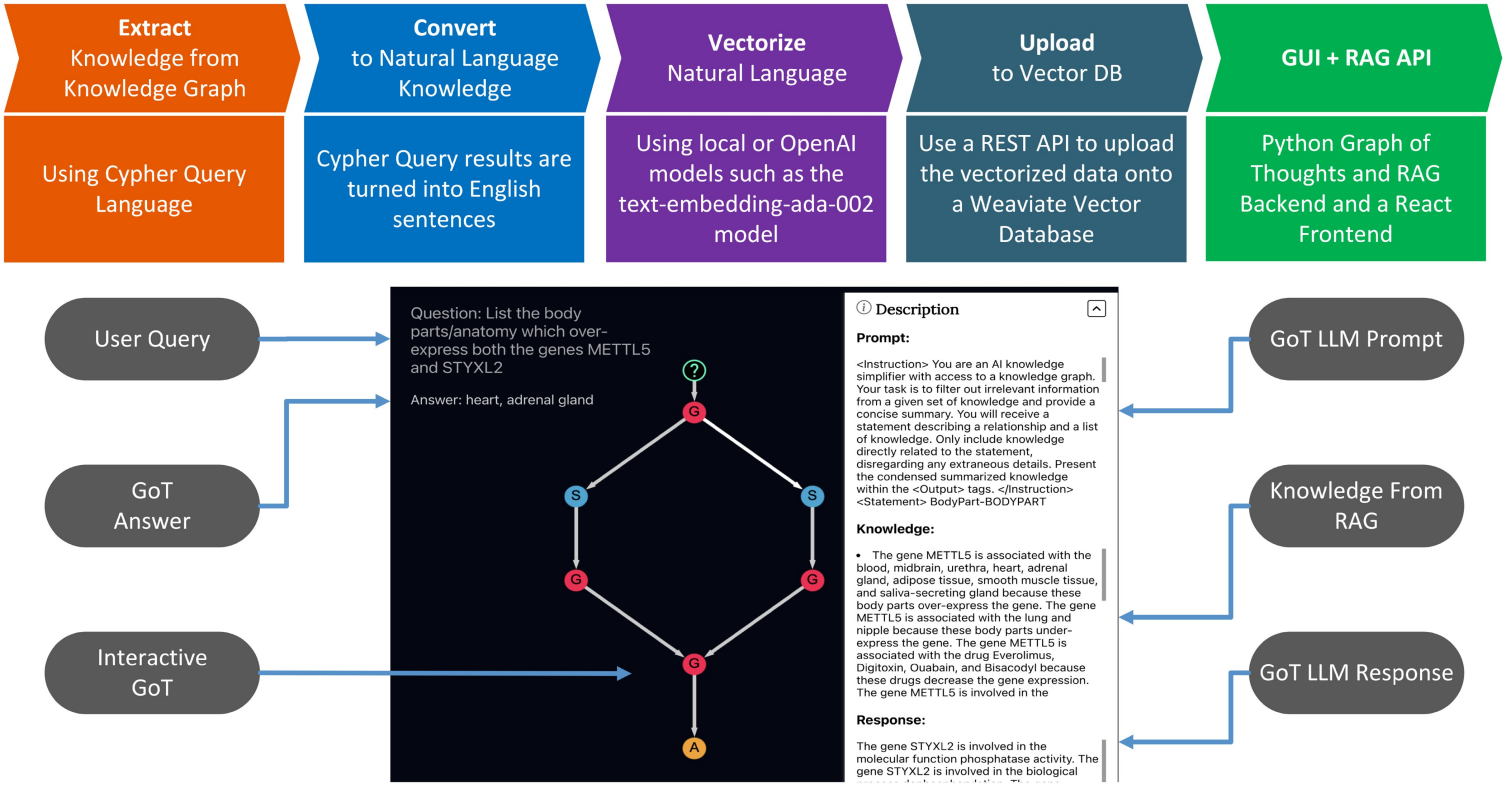
Application flow chart (above) from extraction of knowledge from a knowledge graph, to converting the knowledge graph dump into a list of natural language statements, vectorizing the knowledge and uploading to a vector database, and deploying the KRAGEN GUI (bottom) where the user can ask elaborate questions and view the Graph-Of-Thoughts prompting to view the intuition and knowledge used to solve the problem.

One of the main challenges of using large language models (LLMs) for natural language generation is ensuring the quality and reliability of the generated texts while appreciating the reasoning capabilities of LLMs (Kojima *et al.* 2022). Recent studies on prompting techniques has shown that LLM’s reasoning powers can be further enhanced by chaining the thoughts into a flowchart like structure (Wei *et al.* 2022). While LLMs can produce fluent and coherent texts, without proper vetting, they may lack factual consistency and may introduce irrelevant or false information that is not supported by evidence (Ji *et al.* 2023). A common approach to address this issue is to augment the LLMs with external knowledge sources, such as knowledge graphs or relational databases, that can provide relevant facts and context for the generation task (Lewis *et al.* 2020). This approach is known as Retrieval Augmented Generation (RAG), and it has been shown to improve the performance of LLMs on various knowledge-intensive tasks (Wang *et al.* 2019, Brate *et al.* 2022). However, RAG also has some limitations, such as the difficulty of selecting the most appropriate knowledge points from a large and noisy knowledge source through methods like vector similarity search, and the lack of transparency and explainability of the LLM’s reasoning process. In this paper, we propose a novel open source application, Knowledge Retrieval Augmented Generation ENgine (KRAGEN), that aims to overcome these limitations by using a graph-of-thoughts (GoT) technique to model and execute complex problems with LLMs (Besta *et al.* 2023). GoT is a framework that allows us to decompose a problem into subproblems, and to represent the information generated by the LLM as a graph, where vertices are units of information and edges are dependencies. GoT enables us to perform various graph like operations such as creating edges and joining thoughts to self-loop operation of improving vertex thought quality. Since a GoT is necessarily a graph, KRAGEN can provide a visual interface that shows the LLM’s thought process, revealing how it constructs and validates its answer using transitive logic and factual evidence. The novelty in combining the RAG framework with GoT, by using a graph structure for navigating thoughts of quality knowledge, we can achieve more explainable and trustworthy natural language generation (Lv *et al.* 2019) (Fig. 1).

## 2 Application features

KRAGEN embeds the knowledge graph information into vector embeddings to create a searchable vector database. This database serves as the backbone for the RAG system, which retrieves relevant information to support the generation of responses by a language model. Once the database is set, the GUI for KRAGEN will allow the user to interact and ask complex questions regarding the knowledge graph for higher quality answers (Yasunaga et al. 2021). To do this, we must follow these steps:

### 2.1 Knowledge graph embedding with Weaviate

The first step of KRAGEN is to embed the knowledge graph into a vector space, where each entity and relation is represented by a vector. To do this, we use Weaviate, an open source vector database that supports various embedding models and query methods (https://github.com/weaviate/weaviate).

One of the main advantages of Weaviate is that it supports hybrid search, which combines the best of keyword and vector search. Hybrid search can improve the accuracy and relevance of the search results, by using both the semantic similarity and the keyword frequency of the query and the documents. This feature is very useful for our application, as it enables us to retrieve the most appropriate knowledge points for the generation task given that we can break down a query into parts that can hone in on a specific keyword.

KRAGEN simplifies this process by having the user run a “docker compose” command that converts the knowledge graph dump into natural language, embeds the texts into vectors using an embedding model, and uploads the vectors into the Weaviate database deployed by Docker. The conversion details from Alzheimer’s knowledge graph (AlzKB) to weviate are provided in the GitHub repository. This process can be universally applied to data extracted from any other source, structured or unstructured.

### 2.2 Graph of thoughts prompting

The graph of thoughts (GoT) is a technique that allows us to solve elaborate problems with LLMs. Recent studies of GoT show significant improvements compared to that of zero shot or few shot prompting (Besta *et al.* 2023). GoT decomposes a problem into subproblems, and to represent the information generated by the LLM as a graph, where vertices are thoughts and edges are dependencies. By using GoT, we can control and guide the LLM’s generation process, and achieve more explainable and trustworthy natural language generation.

KRAGEN’s GoT system works as follows: given a user query, KRAGEN parses the query and generates a graph structure plan, which defines the vertices and edges of the GoT. Each vertex corresponds to a subproblem that the LLM needs to solve, and each edge corresponds to a dependency that the LLM needs to follow. KRAGEN then executes the graph structure plan, by generating a natural language prompt for each vertex, and feeding the prompt to the LLM. The LLM then uses the RAG system to retrieve relevant information from the vector database, and generates a natural language response for each thought vertex that requires exact knowledge to resolve the prompt. The response is then used as a thought, which can be fed to other vertices as inputs, or returned to the user as outputs. The novel use of combining GoT with the RAG framework enables to user to ask and navigate through complex problems while still being able to understand the intuition behind the decision making made by the LLM.

### 2.3 GoT visualization

KRAGEN’s graphical user interface (GUI) is designed to facilitate the interaction between the user and the LLM, visualizing the GoT and its respective thought nodes. The GUI is implemented using React and communicates with a Python backend that handles the LLM and the database queries. The GUI consists of three main components: a query box, a GoT panel, and a description box. The query box allows the user to enter a natural language question or a problem statement that they want the LLM to answer or solve. The GoT panel displays the GoT in response to the user’s query, using a graph layout that shows the vertices and edges of the GoT, as well as the labels and attributes of each vertex. The user can interact with the GoT panel by clicking on any vertex to see more details within the description box, such as the prompt of the node, the knowledge pulled from the RAG, and the answer the LLM provided which will be fed downstream into further GoT nodes until a solution is arrived.

## 3 Example use case: Alzheimer’s disease knowledge graph

To demonstrate the practicality and versatility of KRAGEN, we present a use case involving a publicly available Alzheimer’s knowledge graph, AlzKB (https://alzkb.ai/). AlzKB comprises comprehensive data on genes, diseases, drugs, and other relevant entities, along with the relationships between these nodes (Romano *et al.* 2023). We utilized a Neo4j database dump, which includes both node properties and the edges connecting them, to create an enriched vector database for KRAGEN.

### 3.1 Data preparation

The AlzKB Neo4j knowledge base dump was processed to generate vector embeddings for all the nodes and relationships. This step involved encoding the properties of genes, diseases, drugs, and other entities into a vector space, and similarly transforming the relationships into vectors that preserve the semantic context of the graph structure.

### 3.2 RAG deployment

Once the vectors were generated, KRAGEN will deploy a Weaviate server and upload the vector data. The prompts within KRAGEN must be adjusted on domain-specific language to increase the accuracy of responses related to Alzheimer’s research. The RAG system was configured to utilize the vector embeddings to retrieve information relevant to user queries, emulating the capabilities of native Neo4j Cypher queries through natural language interactions.

### 3.3 Query processing and response generation

When a user poses a question to the KRAGEN such as “Which drugs bind to the genes APOE and PLAU?,” KRAGEN leverages the language model to understand the query context, develops a graph structure plan to answer the question by reducing the question into subproblems, and run the RAG system to search the vector database for each node requiring data. The retrieved vectors correspond to the knowledge graph entities and relationships that best match the divided query. In the above sample question, the GoT will create two separate thought nodes searching knowledge for “Drug-CHEMICAL BINDS GENE-APOE” and “Drug-CHEMICAL BINDS GENE-PLAU” using the RAG. Without the GoT’s advanced prompting, a simple RAG pull of knowledge using the original question will introduce a lot of noisy data points, which will provide the LLM with too much to work with. By dividing the problem into key parts dynamically, we can refine the RAG by targeting simpler relationships. KRAGEN’s GoT then synthesizes this information into a final node, providing an answer to the user.

### 3.4 Results

To test the performance of KRAGEN, we implemented an input/output style of prompting, which is a common implementation involving RAG and inserts the knowledge directly as context. After comparing the performance between input/output prompting and the GoT both using the same RAG framework, we saw an improvement on multiple choice questions and true or false questions which is a similar improvement compared to other literature GoT benchmarks (Besta *et al.* 2023). Concerning multiple choice questions, the accuracy of 498 1-hop reasoning and 419 2-hop reasoning improved from 56.6% to 70.4% and 53.1% to 71.8%, respectively. As for the accuracy of true or false questions, 560 1-hop reasoning improved from 68.6% to 80.3%, however for the 540 2-hop reasoning questions, we noticed only a slight improvement of 62.4%–62.9%. In LLMs, the choice of training prompts significantly impacts performance outcomes. However, our primary focus lies in evaluating the performance improvements attributed to the Graph of Thoughts (GoT) strategy, rather than delving into complex prompt engineering. As a result, we adopted a consistent prompt format across all question types, rather than fine-tuning prompts individually. With more tweaking of the prompts and more few shot examples within the prompts, we are certain that the scores would increase. The full transparency of the decision-making through KRAGEN’s visualization provides the user a more symbiotic partnership with AI to aid in complex problem-solving. In our study, we also conducted a performance comparison between KRAGEN and baseline GPT models, including a well-known biomedicine-specialized LLM called BioGPT (Luo et al. 2022) and a robustly designed LLM known as OpenChat (Wang *et al.* 2023). Notably, KRAGEN demonstrated superior performance in this evaluation. Full details and experiments are provided in the Github repository.

## 4 Conclusion

In this article, we introduced KRAGEN that combines the reasoning power of LLMs and the factual feedback of knowledge graphs to answer complex questions and problems. KRAGEN uses an advanced prompting technique called graph of thoughts to model and execute the reasoning process of the language model, and provides a visual interface that shows the logic and evidence behind the generated responses. We demonstrated the features and benefits of KRAGEN using a real-world example of an Alzheimer’s disease knowledge graph, AlzKB. We showed how KRAGEN can retrieve relevant facts from the knowledge graph, generate coherent and informative answers, and explain the connections between the genes, diseases, and drugs related to Alzheimer’s. We believe that KRAGEN is a valuable tool for researchers and practitioners who want to use language models for natural language generation, as it can improve the quality, reliability, and explainability of the generated texts.

We can further foresee KRAGEN can inspire future work on developing more advanced and interactive applications that leverage language models and knowledge graphs. In particular, we envision that KRAGEN can be applied to the medical domain, where doctors can connect patient data and biomedical resources into the RAG system, and use KRAGEN to navigate complex problems and provide personalized and evidence-based solutions with full transparency of reasoning and knowledge. Our advanced data conversion process ensures compatibility for handling various biomedical information from different sources, whether structured or unstructured. Additionally, leveraging Docker’s inherent scalability, our system supports deployment on robust computational platforms. This strategic design choice enables KRAGEN to handle the intricate integration processes associated with knowledge graphs and extensive biomedical datasets.
